# Recent hydromorphone injection and risk of incident infective endocarditis among people who inject drugs in Toronto, Canada: a cohort study

**DOI:** 10.1186/s12954-026-01479-x

**Published:** 2026-06-20

**Authors:** Nicholas Bakewell, Zachary Bouck, Zoë R. Greenwald, Katie Upham, Christine M. Warren, Marina G. Birck, Cristiano S. Moura, Sasha Bernatsky, Autumn Neville, Tara Gomes, Daniel Werb

**Affiliations:** 1 Centre on Drug Policy Evaluation, St. Michael’s Hospital, Unity Health Toronto, 209 Victoria St, 3rd Floor, Toronto, ON M5B 1T8 Canada; 2 ICES, Toronto, ON Canada; 3 Dalla Lana School of Public Health, University of Toronto, Toronto, ON Canada; 4 Centre for Outcomes Research and Evaluation (CORE), The Research Institute of the McGill University Health Centre, Montreal, QC Canada; 5 Department of Epidemiology, Biostatistics and Occupational Health, McGill University, Montreal, QC Canada; 6 Institute of Health Policy, Management and Evaluation, University of Toronto, Toronto, ON Canada; 7 Leslie Dan Faculty of Pharmacy, University of Toronto, Toronto, ON Canada; 8 MAP Centre for Urban Health Solutions, St. Michael’s Hospital, Unity Health Toronto, Toronto, ON Canada; 9 Division of Infectious Diseases and Global Public Health, University of California San Diego, La Jolla, CA USA

**Keywords:** Cohort study, People who use drugs, Infective endocarditis, Hydromorphone

## Abstract

**Background:**

People who inject drugs (PWID) are at increased risk of infective endocarditis (IE). While opioid injection is a major risk factor for IE, the risk associated with specific prescription opioids, such as hydromorphone, remains unclear and is inconsistently reported, despite a hypothesized pathway whereby the insolubility of controlled-releaseformulations may promote injection equipment reuse and bacterial contamination. Therefore, we estimated and compared the 1-year incidence rates of IE among PWID reporting injection of hydromorphone versus non-hydromorphone opioid medications and versus other opioids/drugs.

**Methods:**

Baseline questionnaire data on PWID in the Ontario Integrated Supervised Injection Services cohort (November 2018–March 2020) were linked to health administrative databases. IE incidence was calculated over 1-year post-baseline. Participants were followed until first of IE, death, or 1-year censoring. Exposure was self-reported recent (past 6 months) injection of: hydromorphone, non-hydromorphone opioid medications of interest, or other opioids/drugs. Cox regression estimated associations, adjusting for age, gender, ethnicity, injection drug use, and prior injection-related infections.

**Results:**

Among the 514 participants included, 191 reported recent injection of hydromorphone, 32 non-hydromorphone opioid medications of interest, and 291 other opioids/drugs. IE incidence rates were low across groups: 1.6, 3.3, and 1.8 per 100 person-years. Adjusted hazard ratio comparing recent hydromorphone injection versus non-hydromorphone opioid medications of interest was 0.64 (95% confidence interval (CI) 0.06–6.53); versus other opioids/drugs was 0.82 (95% CI 0.19–3.51).

**Conclusions:**

In this cohort of PWID, no clear association was found between hydromorphone injection and IE when compared to non-hydromorphone opioid medications or to other opioids/drugs. Due to low event rates and the resulting wide CIs, the findings are limited by low statistical precision. Further research with larger cohorts is warranted to quantify IE risk of specific injected drugs among PWID.

**Supplementary Information:**

The online version contains supplementary material available at https://doi.org/10.1186/s12954-026-01479-x.

## Background

The opioid crisis remains a significant public health emergency in North America. While modes of non-medical opioid administration are reportedly shifting to increased non-injection use (e.g., inhalation), injecting remains a high-risk route of administration for a range of health harms, including infective endocarditis (IE) [1–6]. IE is a life-threatening infection of heart valves, that has been rising in incidence among people who inject drugs (PWID) [7, 8]. Beyond frequency of injection and substances used, commonly reported risk factors for IE among PWID include unsterile injection practices, demographic factors such as younger age, and social–structural factors such as insufficient housing [7, 9–11]. In Toronto, Canada, harm reduction services, including supervised consumption services, needle and syringe programs, and opioid agonist treatment (OAT), have been available and expanding since 2017, providing PWID with access to sterile injection equipment and related supports [12].

In Ontario, Canada, some evidence exists suggesting a correlation between increased hydromorphone prescribing rates and increased IE incidence among PWID, both at the population [8] and individual [13] levels. At the population level, following the withdrawal of controlled-release oxycodone from the Canadian market in the fourth quarter of 2011, Ontario’s prescribing market shifted markedly, with hydromorphone’s share of all opioid prescriptions rising from 16% in April 2006 to 53% in September 2015 [8]. While this market shift has not been directly linked to rising IE incidence among PWID, it has been hypothesized to play a role [8]. At the individual level, it has been hypothesized that injecting controlled-release hydromorphone (which is prescribed primarily as oral capsules) may elevate the risk of IE and transmission of blood-borne infections [7, 14]. This elevated risk is attributed to the insolubility of controlled-release hydromorphone, which can lead to residue buildup on drug preparation equipment, incentivizing equipment reuse and promoting growth of bacteria and other organisms [7, 15, 16]. Within the context of harm reduction, this suggests that the rising prevalence of controlled-release hydromorphone in Ontario may necessitate specific interventions, such as targeted education regarding the risks of equipment reuse [7]. However, a recent systematic review concluded that there exists low-quality and scant real-world evidence for this causal pathway, leaving substantial uncertainty about whether and how controlled-release hydromorphone may influence the risk of IE and the transmission of blood-borne infections [17].

Given the ongoing opioid crisis and rise in hydromorphone prescribing, coupled with previous evidence suggesting links to IE among PWID, there is a pressing need for further research into this issue. The objectives of this study were therefore to (1) describe the characteristics of PWID who are at risk for incident IE, and (2) compare rates of incident IE among PWID self-reporting injection of hydromorphone relative to other opioids or drugs.

## Methods

### Study design and participants

The Ontario Integrated Supervised Injection Services cohort (OiSIS) is an open, prospective cohort study of PWID in Toronto, Canada [12], ongoing under the name Toronto Disparities, Overdose, and Treatment (T-DOT) study. Recruitment started 5 November 2018 through self-referral, snowball sampling, and community or street outreach [12]. At baseline, participants were at least 18 years old, English-speaking, living in Toronto, reported injecting drugs in the past 6 months, provided informed consent, and completed an interviewer-administered questionnaire [12].

Data on sociodemographic characteristics, drug use behaviors, use of harm reduction services, and treatment for substance use disorders were collected at the baseline interview using interviewer-administered questionnaires [12]. Participants were also asked for consent to have their baseline questionnaire data linked with health administrative databases at ICES (formerly known as the Institute for Clinical Evaluative Sciences) [12]. ICES is an independent, non-profit research institute whose legal status under Ontario’s health information privacy law allows it to collect and analyze health care and demographic data, without consent, for health system evaluation and improvement.

Baseline questionnaire data were linked to the following health administrative databases held at ICES: Registered Persons Database (RPDB; demographic information and vital statistics for Ontario Health Insurance Plan-eligible Ontario residents); Discharge Abstract Database (DAD; procedures and diagnoses for acute inpatient hospital admissions); the National Ambulatory Care Reporting System (NACRS; procedures and diagnoses for emergency department visits); Ontario Drug Benefit (ODB) Database (prescription drug claims from community pharmacies for individuals covered under ODB public drug plan); and Narcotics Monitoring System (NMS; prescriptions for controlled substances dispensed at community pharmacies in Ontario, irrespective of payer). These datasets were linked using unique encoded identifiers and analyzed at ICES. Full details of this linkage have been previously published [18].

To be eligible for the present study, participants had to complete a baseline questionnaire between 5 November 2018 and 19 March 2020, before a temporary pause in enrolment due to COVID-19 restrictions; consent to having their baseline questionnaire data linked at ICES; and not been hospitalized for IE (defined as a hospitalization record with any of the following ICD-10 codes in any position: I330, I339, I38, I39 or B376) within the past 6 months before the baseline interview date.

### Variable definitions

#### Outcome

The primary outcome was time to first IE hospitalization, within the first year of study enrolment. The index date for our cohort was the date of the baseline interview. Follow-up time continued until the first of IE event of interest, death, or the censoring date of 1 year after a participant’s baseline interview date.

#### Exposure

Three exposure groups using self-reported questionnaire data collected at baseline were defined: recent (within past 6 months before baseline) injection of hydromorphone (including oral and injectable controlled- and immediate-release hydromorphone); recent injection of non-hydromorphone opioid medications of interest (oxycodone hydrochloride; oxycodone hydrochloride and acetaminophen; or morphine); and recent injection of other opioids or drugs (excluding hydromorphone and non-hydromorphone opioid medications of interest). The exposure groups were created hierarchically: the subset of participants reporting recently injecting hydromorphone were first classified, then those reporting recently injecting non-hydromorphone opioid medications of interest, with the remaining participants being classified as having a recent injection of other opioids or drugs. Of note, participants were not excluded based on their ongoing use of unregulated drugs (e.g., illegally-manufactured fentanyl and other opioid analogues, benzodiazepines, stimulants, or sedatives). As such, the exposure categories should be understood as the combined exposure of drugs specific to each category as well as the participant’s use of other unregulated drugs. Non-hydromorphone opioid medications of interest were selected as an active comparator group of other prescription opioids commonly injected by participants, enabling comparison of hydromorphone-specific risk while reducing confounding by factors common to injection drug use more broadly. The third group, recent injection of other opioids or drugs, served as a broader comparator encompassing participants injecting unregulated substances such as fentanyl or heroin, allowing for the contextualization of hydromorphone-associated IE risk against general injection drug use.

While hydromorphone dispensations among participants can be measured in the NMS database, the exposure groups were defined using only (self-reported) baseline questionnaire data collected on measures of recent injection of opioid medications, as participants may inject diverted medications (i.e., medications not prescribed to them) which would not be captured in their NMS dispensation data. The accuracy of self-reported data was also considered, noting that a previous validation study concluded that self-reported OAT measures are generally accurate among PWID [18].

#### Baseline characteristics

Baseline characteristics (ascertained as of the date of the baseline interview) included: age (in years; data source: RPDB), time since first injection (in years; baseline questionnaire), ethnicity (white vs. non-white; baseline questionnaire), cisgender man (yes or no; baseline questionnaire), recent income from sex work (yes or no; baseline questionnaire), recent frequency of injection drug use (daily, weekly or less than weekly; baseline questionnaire), recent OAT (yes or no; NMS), recent supervised injection service use (yes or no; baseline), recent opioid-related overdose [ICD-10 codes: T400, T401, T402, T403, T404, or T406; DAD (admission diagnosis) and NACRS (any diagnosis)]; recent incarceration (yes or no; baseline), recent hospitalization (yes or no; DAD), recent frequency of ED visits (NACRS), recent hospitalization for serious injection-related infection [excluding IE; admission diagnosis coded as any of the following ICD-10 codes: M86 or M00 (non-vertebral bone infection); G061, M462, M463, M464, or M465 (spinal infection); L03, L02, or M762 (skin or soft tissue infection); DAD]. We adapted these definitions from a previously published study that explored inpatient hospitalizations for serious infections among people with opioid use disorder [19]. The lookback period for baseline characteristics retrieved from health administrative databases was 6 months (DAD, NACRS, and NMS), and the same for variables retrieved from the baseline questionnaire classified as recent. Concurrent injection drug use at baseline (recent injection of morphine, oxycodone hydrochloride, oxycodone hydrochloride and acetaminophen, heroin, fentanyl, amphetamine-type stimulants, crack/rock cocaine, benzodiazepines, and prescription stimulants) was additionally captured and is reported in Appendix Table A1.

### Statistical analysis

Participants’ baseline characteristics were summarized using frequencies (percentages) for categorical variables and median [interquartile ranges (IQRs)] for continuous variables, overall and stratified by exposure group. Categorical variables were compared across exposure groups using a Chi-squared test, or a Fisher’s exact test when expected cell counts were less than 5, and continuous variables were compared using a Kruskal–Wallis test.

The crude event-free probability within 1 year of baseline for incident IE was estimated using the Kaplan–Meier method and differences were tested between exposure groups using the log-rank test.

The crude and adjusted associations between the exposure of interest and incident IE were estimated using Cox proportional hazards regression; focusing on contrasts with the recent injection of hydromorphone group. In the multivariable Cox regression analyses, we adjusted for potential confounding variables of the relationship between the exposure of interest and incident IE, as informed by previous studies: age (continuous) [20], being a cisgender man [7, 11], ethnicity [21], recent frequency of injection drug use [7], and recent hospitalization for injection-related infection (excluding IE) [20, 22].

Any cells with a count between one and five were suppressed per ICES data privacy rules, with suppression of cells for other levels of the same variable (through use of ranges)—as needed—to prevent back calculation of the suppressed small cells and possible re-identification of individual participants. Missing data were handled using listwise deletion, excluding participants who were missing data on any variable included in an analysis.

Statistical significance of regression analyses was defined as a two-tailed *p* value < 0.05. All analyses were performed using SAS Enterprise Guide 7.1 (SAS Institute Inc., Cary, NC). This study followed the REporting of studies Conducted using Observational Routinely-collected health Data (RECORD) [23] reporting guideline, which extends the Strengthening the Reporting of Observational Studies in Epidemiology (STROBE) statement to observational studies that use routinely collected health data (Appendix).

## Results

Among the 701 participants recruited to the OiSIS cohort between 5 November 2018 and 19 March 2020, 632 (90.2%) consented to ICES (health administrative) database linkages. Of those consenting, 521 (82.4%) were successfully linked to ICES databases and were eligible for inclusion. Applying exclusion criteria resulted in a final cohort of 514 participants.

Among the 514 participants, 37.2% reported recent injection of hydromorphone (N = 191 participants), 6.2% non-hydromorphone opioid medications of interest (N = 32 participants), and 56.6% other opioids or drugs (N = 291 participants). Overall, the median age was 40 years (IQR 33–49), with 66.5% cisgender men (N = 342), 55.1% white (N = 283), 56.4% reporting daily injection (N = 290), and 4.7% with a prior injection-related infection (excluding IE) hospitalization within the past 6 months (N = 24). While participants in the three exposure groups generally had similar characteristics at baseline, there were statistically significant between-exposure group differences. Those in the recent injection of hydromorphone and non-hydromorphone opioid medications of interest groups had a higher frequency of injection drug use, compared to those in the other opioids or drugs group (*p* < 0.001). Those in the recent injection of hydromorphone group had a higher proportion of participants reporting recent opioid-related non-fatal overdose relative to those in non-hydromorphone opioid medications of interest and other opioids or drugs groups (*p* < 0.001; Table 1). Concurrent injection drug use by exposure group is presented in Appendix Table A1; polysubstance injection was near-universal across all groups, with statistically significant between-group differences observed for heroin, fentanyl, crack/rock cocaine, benzodiazepines, and prescription stimulants.

**Table 1:** Baseline characteristics of people who inject drugs, included in analyses to assess incident infective endocarditis

Baseline characteristics	Overall	Exposure group			*p* value
		Recent injection of hydromorphone^a^	Recent injection of non-hydromorphone opioid medications of interest^b^	Recent injection of other opioids or drugs^c^	
Number of participants	514	191	32	291	
Age (years), median (IQR)	40 (33–49)	39 (33–49)	43 (35–50)	40 (33–50)	0.73
Time since first injection (years), median (IQR)	13 (6–27)	13 (5–28)	12 (6–28)	13 (6–26)	0.99
Ethnicity—White, n (%)	283 (55.1%)	112 (58.6%)	21 (65.6%)	150 (51.5%)	0.15^FE^
Missing	1	1	0	0	
Cisgender man—Yes, n (%)	342 (66.5%)	125 (65.4%)	20 (62.5%)	197 (67.7%)	0.61^FE^
Missing	10	6	0	4	
Recent income from sex work—Yes, n (%)	159 (30.9%)	63 (33.0%)	8 (25.0%)	88 (30.2%)	0.58^FE^
Missing	3	0	0	3	
Recent frequency of injection drug use, n (%)					< 0.001^FE^
Less than weekly	80 (15.6%)	13 (6.8%)	6 (18.8%)	61 (21.0%)	
Weekly	144 (28.0%)	52 (27.2%)	8 (25.0%)	84 (28.9%)	
Daily	290 (56.4%)	126 (66.0%)	18 (56.3%)	146 (50.2%)	
Recent opioid agonist treatment—Yes, n (%)	264 (51.4%)	110 (57.6%)	15 (46.9%)	139 (47.8%)	0.10
Recent opioid-related overdose—Yes, n (%)	78 (15.2%)	46 (24.1%)	1–5 (3.1–15.6%)*	26–30 (8.9–10.3%)*	< 0.001
Recent supervised injection service use—Yes, n (%)	433 (84.2%)	164 (85.9%)	28 (87.5%)	241 (82.8%)	0.58
Recent incarceration-Yes, n (%)	191 (37.2%)	81 (42.4%)	7 (21.9%)	103 (35.4%)	0.12
Missing	44	16	5	23	
Recent hospitalization—Yes, n (%)	82 (16.0%)	32 (16.8%)	1–5 (3.1–15.6%)*	46–50 (15.8–17.2%)*	0.83
Recent hospitalization for serious injection-related infection^d^—Yes, n (%)	24 (4.7%)	8 (4.2%)	1–5 (3.1–15.6%)*	11–15 (3.8–5.2%)*	0.35^FE^
Recent frequency of ED visits, median (IQR)	2 (0–3)	2 (0–4)	2 (0–2)	1 (0–3)	0.05

*IQR* interquartile range, *ED* emergency department

*Exact value suppressed due to small cells (per ICES data privacy rules). ‘Recent’ denotes activities, experiences, and/or behaviours within the past 6 months

^a^Recent (past 6 months) injection of hydromorphone (including oral and injectable controlled release and immediate release hydromorphone)

^b^Recent injection of non-hydromorphone opioid medications of interest (oxycodone hydrochloride; oxycodone hydrochloride and acetaminophen; or morphine)

^c^Recent injection of other opioids or drugs (excluding hydromorphone and other non-hydromorphone opioids of interest)

^d^Due to eligibility criteria for cohort, this excludes recent hospitalizations for infective endocarditis (as all participants did not have a recent IE hospitalization) but includes recent hospitalizations for skin and soft tissue infections, non-vertebral bone infections, and/or spinal infections

^FE^Fisher’s exact test used due to small expected cell sizes less than 5

**Fig. 1:**
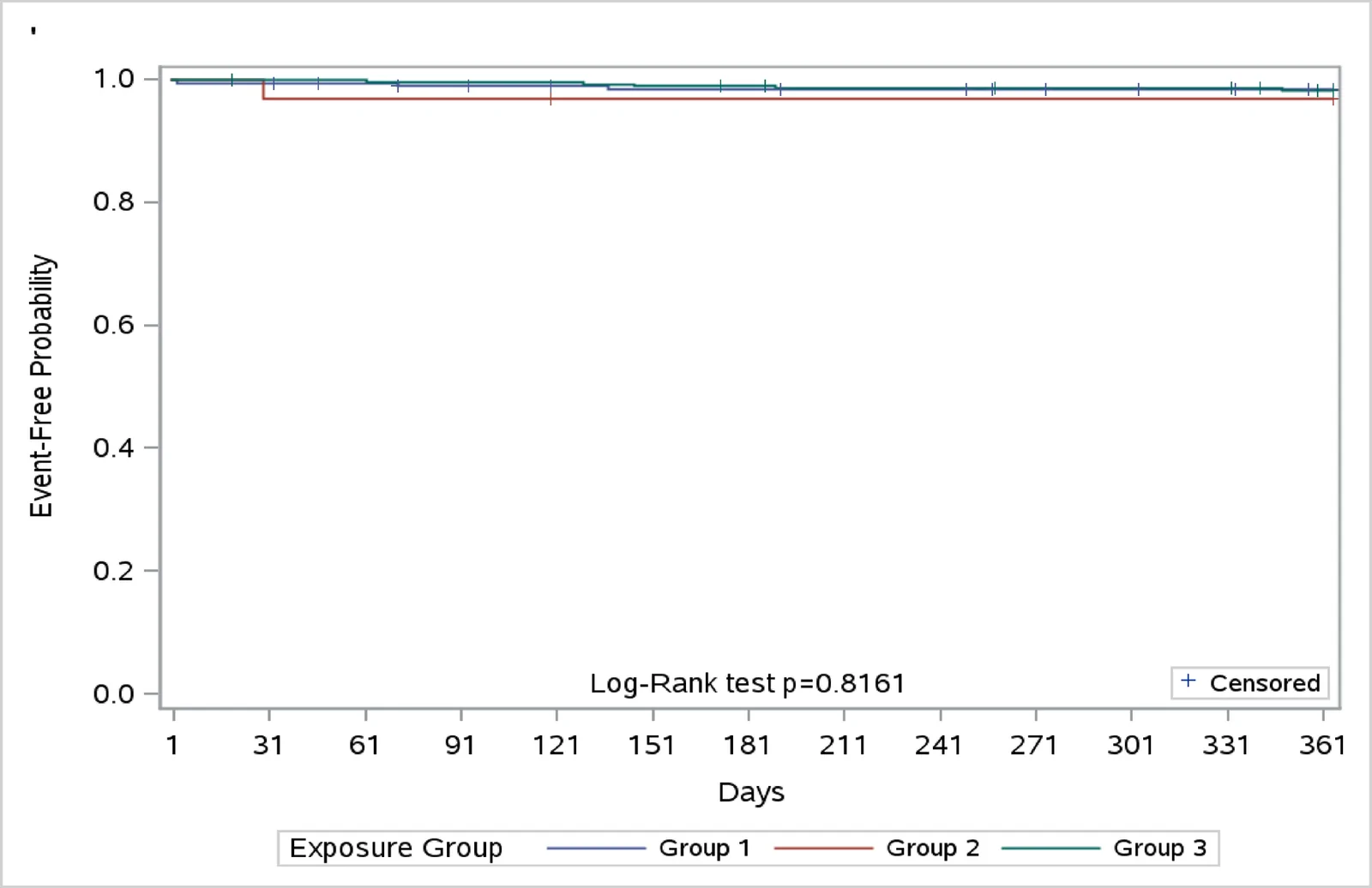
Kaplan–Meier plot demonstrating (cumulative) event-free probability of incident infective endocarditis by duration of follow-up in the first year after baseline interview by exposure group. *Notes*: Group 1: Recent (past 6 months) injection of hydromorphone (including oral and injectable controlled release and immediate release hydromorphone); Group 2: Recent injection of non-hydromorphone opioid medications of interest (oxycodone hydrochloride; oxycodone hydrochloride and acetaminophen; or morphine); Group 3: Recent injection of other opioids or drugs (excluding hydromorphone and other non-hydromorphone opioids of interest)

The incidence rates of IE were 1.6, 3.3, and 1.8 per 100 person-years for participants reporting recent injection of hydromorphone, non-hydromorphone opioid medications of interest, and other opioids or drugs, respectively (Table 2). Figure 1 presents Kaplan–Meier survival curves for 1 year of follow-up by exposure group within our cohort. The log-rank test did not indicate a statistically significant difference in the crude event-free probability within 1 year of baseline for incident IE across the exposure groups (*p* = 0.82). After adjustment for covariates (age, being a cisgender man, ethnicity, recent frequency of injection drug use, and recent hospitalization for serious injection-related infection), the adjusted hazard ratio (HR) comparing incident IE in those reporting recent injection of hydromorphone versus non-hydromorphone opioid medications of interest was 0.64 (95% CI 0.06–6.53). The adjusted HR comparing incident IE in those reporting recent injection of hydromorphone versus other opioids or drugs was 0.82 (95% CI 0.19–3.51) (Table 2).

**Table 2:** Unadjusted and adjusted hazard ratios for the association between exposure of interest and infective endocarditis, with 95% confidence intervals

Contrast		Incidence rate (# of events per 100 person-years)				Unadjusted model (N = 514)		Adjusted model^b,c^ (N = 502)	
Exposure of interest	Reference	Exposure		Reference		Hazard ratio	95% Confidence interval	Hazard ratio	95% Confidence interval
		Event count^a^	Incidence rate	Event count^a^	Incidence rate				
Recent injection of hydromorphone	Recent injection of non-hydromorphone opioid medications of interest	1–5	1.6	1–5	3.3	0.50	0.05–4.77	0.64^c^	0.06–6.53^c^
Recent injection of hydromorphone	Recent injection of other opioids or drugs	1–5	1.6	1–5	1.8	0.93	0.22–3.90	0.82^c^	0.19–3.51^c^

‘Recent’ denotes activities, experiences, and/or behaviours within the past 6 months

^a^Any cells with a count between one and five were suppressed per ICES data privacy rules, with suppression of cells for other levels of the same variable (through use of ranges)—as needed—to prevent back calculation of the suppressed small cells and possible re-identification of individual participant

^b^Analysis restricted to complete cases (those with non-missing outcome, exposure, and covariate data)

^c^Model adjusted for the following baseline characteristics (included as covariates): age (in years; continuous), being a cisgender man (yes or no), ethnicity (White; yes or no), recent frequency of injection drug use (daily, weekly, less-than-weekly), and recent hospitalization for serious injection-related infection (excluding infective endocarditis; yes or no)

## Discussion

Using self-reported baseline questionnaire data from a cohort of PWID in Toronto (Ontario, Canada) linked to health administrative databases, no clear association was found between recent injection of hydromorphone and incident IE within a 1-year follow-up period. These findings contribute to the existing evidence regarding the association between hydromorphone and incident IE among PWID. Specifically, by leveraging linkages between primary questionnaire data and health administrative databases, a key contribution of this study is reporting characteristics of PWID at risk for IE, as presented in Tables 1 and A1, which have been missing from prior reports, and which may have confounded the previously-identified associations between hydromorphone prescription and IE incidence.

A previous Ontario-based population cohort study reported a statistically significant association between hydromorphone opioid medication exposure and increased IE risk among PWID, with those prescribed controlled-release hydromorphone having over three times the odds of developing IE compared to those prescribed non-hydromorphone opioid medications [adjusted odds ratio: 3.3 (95% CI 2.1–5.6)] [13]. This contrasts with our findings, which did not establish this association (though this may be due to the limited precision of our estimates stemming from low event rates). It is noteworthy that we included all hydromorphone formulations in our recent injection of hydromorphone exposure group, which limited our ability to distinguish the associations of controlled-release and other formulations of hydromorphone with incident IE. Consequently, our findings represent a mixture of effects for different hydromorphone formulations, some of which may have a stronger association with IE than others, diluting the observed effect, potentially biasing our results towards the null.

Moreover, there are differences between our study and the previous study investigating the association between hydromorphone prescription and IE incidence [13] in study design, follow-up duration and sample size. For example, time trends during the long period (April 2006–September 2015) of the previous study may have introduced significant (time-dependent) confounding bias [13]. Therefore, future studies should carefully consider potential time trends in the exposure and outcome, especially those that may be influenced by drug market shifts and other trends related to the intersection of the opioid crisis and poverty (e.g., lack of access to sterile injecting equipment or private venues within which to inject), in addition to potential unmeasured confounding as acknowledged by the authors of the previous study [13].

Importantly, our study period overlapped with initial warnings from Weir and colleagues in January 2019 [8] regarding the potential increased IE risk associated with injection drug use and hydromorphone prescriptions in Ontario, and coincided with increased implementation of harm reduction strategies, including targeted safer injection education, and increased access to OAT. This temporal overlap, combined with the widespread availability of these services in Toronto, may have mitigated the risk of IE in our cohort, contributing to the observed low incidence rates.

Finally, it is crucial to acknowledge that IE is a complex, multi-factorial condition. Factors such as frequent injection drug use, unsterile injection practices, and social–structural factors including insufficient housing, play significant roles in IE among PWID [7, 9, 10]. The absence of a clear association in our study does not equate to evidence of no association and underscores the need for future research to explore these complex, multicausal pathways beyond hydromorphone injection, which was beyond the scope of our study.

### Limitations

Despite the valuable insights gained from this study, several limitations must be acknowledged. First, we had limited sample size and low incidence rates to produce precise estimates, and to explore associations specifically for the recent injection of controlled-release hydromorphone. Thus, our findings pertain to the recent injection of any formulation of hydromorphone, and although still informative, should not be interpreted as applying to controlled-release hydromorphone alone. Moreover, as exposure was defined using baseline questionnaire data capturing recent injection within the 6 months prior to cohort entry, we were unable to examine the relationship between duration or time since first hydromorphone injection and incident IE risk. Second, we caution against over-interpreting results due to unmeasured confounding. For example, we did not account for social factors including housing status and access to care [22]; drug-related risk factors for IE such as use of non-sterile syringes and receptive sharing of injection drug use equipment [16]; or vein loss, which may influence both injection site practices and drug formulation preferences over time [24]. Additionally, as the exposure groups reflect each primary exposure drug in combination with participants’ broader patterns of concurrent drug use, any statistically significant association, had one been observed, could not have been attributed to hydromorphone injection alone. Third, our study relies on a convenience sample of PWID living in a large urban centre with access to supervised consumption services, who agreed to participate and be linked to health administrative data, and therefore, our results may not be fully generalized to all PWID, or to settings with lower levels of harm reduction service coverage. Fourth, our study’s follow-up period overlapped with the onset of the COVID-19 pandemic, which may have resulted in an under-diagnosis of IE due to disruptions in healthcare access and utilization. Fifth, we used self-reported data for some variables which may have introduced recall or social desirability bias; however, previous research from this study has shown a very high concordance between self-reported and administratively-captured data [18]. Finally, we used health administrative data to define IE, without requiring clinical confirmation (via chart review or lab testing). However, validation studies have shown hospital and physician billing data can capture IE with high sensitivity (over 90%) [25].

## Conclusions

Among a sample of PWID living in a large urban centre in Canada, we did not observe a clear increased risk of IE associated with recent injection of various hydromorphone formulations after adjusting for a range of socioeconomic, sociodemographic, and drug-related factors. This adds to the literature, which includes past research (based exclusively on health administrative data) that observed an association between hydromorphone prescription and IE incidence. Although no association was found in our study, IE remains a serious and potentially life-threatening complication among PWID, and efforts are needed to reduce its incidence among this population at risk. Further research is critically needed to understand the risk factors that increase IE risk among PWID, including drug use behaviours, and barriers to harm reduction programs and other healthcare services that may mitigate IE risk. Specifically, future studies should prioritize exploring the complex interplay of social determinants of health, injection practices, and access to harm reduction services in relation to IE risk.

## Supplementary Information

Below is the link to the electronic supplementary material.


Supplementary Material 1



Supplementary Material 2


## Data Availability

The dataset from this study is held securely in coded form at ICES. While legal data sharing agreements between ICES and data providers (e.g., healthcare organizations and government) prohibit ICES from making the dataset publicly available, access may be granted to those who meet pre-specified criteria for confidential access, available at www.ices.on.ca/DAS (email: das@ices.on.ca). The full dataset creation plan and underlying analytic code are available from the authors upon request, understanding that the computer programs may rely upon coding templates or macros that are unique to ICES and are therefore either inaccessible or may require modification.
