# Supplementary material for: Recent hydromorphone injection and risk of incident infective endocarditis among people who inject drugs in Toronto, Canada: a cohort study
Source: Harm Reduct J. 2026 Jun 20;23:143. doi: 10.1186/s12954-026-01479-x (PMC13531745; doi:10.1186/s12954-026-01479-x)
Supplement: Supplementary file 2 [file 12954_2026_1479_MOESM2_ESM.docx]

| **Appendix Table A1. Concurrent injection drug use at baseline by exposure group, among people who inject drugs included in analyses to assess incident infective endocarditis** | | | | | |
| --- | --- | --- | --- | --- | --- |
| **Baseline characteristics** | **Overall** | **Exposure group** | | | **p-value** |
|  |  | **Recent injection of hydromorphone^a^** | **Recent injection of non-hydromorphone opioid medications of interest^b^** | **Recent injection of other opioids or drugs^c^** |  |
| Number of participants | 514 | 191 | 32 | 291 |  |
| Recent injection of morphine^d^ – Yes, n (%) | 118 (23.0%) | *87-91 | *27-31 | 0 (0.0%) | <0.001^FE^ |
| Recent injection of oxycodone hydrochloride and acetaminophen^d^ – Yes, n (%) | 38 (7.4%) | *33-37 | *1-5 | 0 (0.0%) | <0.001^FE^ |
| Recent injection of oxycodone hydrochloride^d^ – Yes, n (%) | 26 (5.1%) | 20 (10.5%) | 6 (18.8%) | 0 (0.0%) | <0.001^FE^ |
| Recent injection of heroin – Yes, n (%) | 276 (53.7%) | 119 (62.3%) | 17 (53.1%) | 140 (48.1%) | 0.009^FE^ |
| Recent injection of fentanyl – Yes, n (%) | 379 (73.7%) | 155 (81.2%) | 26 (81.3%) | 198 (68.0%) | 0.004^FE^ |
| Recent injection of amphetamine-type stimulants (i.e., including methamphetamine and Amphetamines) – Yes, n (%) | 314 (61.1%) | 124 (64.9%) | 22 (68.8%) | 168 (57.7%) | 0.19 |
| Recent injection of [crack/rock] cocaine – Yes, n (%) | 285 (55.4%) | 117 (61.3%) | 21 (65.6%) | 147 (50.5%) | 0.03 |
| Recent injection of benzodiazepines – Yes, n (%) | 17 (3.3%) | *11-15 | *1-5 | *1-5 | 0.002 |
| Recent injection of prescription stimulants^e^ – Yes, n (%) | 24 (4.7%) | *12-16 | *1-5 | 7 (2.4%) | 0.009^FE^ |
| * = Exact value suppressed due to small cells (per ICES data privacy rules). ‘Recent’ denotes activities, experiences, and/or behaviours within the past six months.  ^a^ Recent (past six months) injection of hydromorphone (including oral and injectable controlled release and immediate release hydromorphone);  ^b^ Recent injection of non-hydromorphone opioid medications of interest (oxycodone hydrochloride; oxycodone hydrochloride and acetaminophen; or morphine).  ^c^ Recent injection of other opioids or drugs (excluding hydromorphone and other non-hydromorphone opioids of interest).  ^d^ Recent injection of morphine, oxycodone hydrochloride, and oxycodone hydrochloride and acetaminophen define the recent injection of non-hydromorphone opioid medications of interest exposure group; the other opioids or drugs exposure group reports zero recent injection of these medications by construction.  ^e^ Any recent injection involving a prescription stimulant, regardless of co-ingestants, was categorized as prescription stimulant use.  ^FE^Fisher’s exact test used due to small expected cell sizes less than 5. | | | | | |
